# Molecular analysis of circulating tumor cells of metastatic castration-resistant Prostate Cancer Patients receiving ^177^Lu-PSMA-617 Radioligand Therapy

**DOI:** 10.7150/thno.44556

**Published:** 2020-06-18

**Authors:** Katharina Kessel, Robert Seifert, Matthias Weckesser, Wolfgang Roll, Verena Humberg, Katrin Schlack, Martin Bögemann, Christof Bernemann, Kambiz Rahbar

**Affiliations:** 1 Department of Nuclear Medicine, University Hospital Münster, Münster, Germany.; 2 Department of Nuclear Medicine, University Hospital Essen, Essen, Germany.; 3 Department of Urology, University Hospital Münster, Münster, Germany.; 4 West German Cancer Center, Münster.

**Keywords:** CTC, PSMA, ^177^Lu-PSMA-617, prostate cancer

## Abstract

**Rationale:** Lu-177-PSMA-617 radioligand therapy (RLT) is currently under approval for treatment of metastatic castration resistant prostate cancer (mCRPC) patients with late stage disease. However, previous studies demonstrated both heterogeneity of prostate specific membrane antigen (PSMA) expression, as well as response to PSMA treatment among mCRPC patients. Thus, there is an unmet need for identifying predictive parametres prior or under PSMA-RLT treatment. We therefore aimed to correlate several clinical and molecular parameters with response to PSMA treatment in a cohort of mCRPC patients undergoing PSMA RLT followed by a detailed analysis of promising candidates.

**Methods:** Nineteen patients, median age 68.8 years (range: 56.9 - 83.3) with mCRPC were included in this study. We performed baseline analysis of clinical parameters based on PSMA PET/CT, (metabolic tumor volume (MTV), total tumor volume (TTV)), serum PSA, ALP, LDH and gene expression analysis of circulating tumor cells (expression of AR full length (AR-FL), AR splice variant 7 (AR-V7), PSA and PSMA) as well as common markers for neuroendocrine differentiation (NED).

**Results:** Patients presented with bone, lymph node, and visceral metastases (89%, 68%, and 21%, respectively). All patients were pretreated with docetaxel, either abiraterone or enzalutamide, or both. Biochemical response in terms of PSA decline ≥50 or ≥30% was observed in 42% and 63%, respectively. There were significant correlations between PSA and PSMA mRNA expression, as well as tumor volumes (both MTV and TTV), AR-FL and AR-V7 mRNA expression. However, there was no correlation with response to PSMA treatment. Furthermore, none of these parameters was significantly correlated with baseline serum PSA values. Common NED markers were shown to be specifically high expressed and revealed impact on OS independent from AR-V7 gene expression.

**Conclusion:** We demonstrate that AR-FL and its splice variant AR-V7 might serve as prognostic biomarkers displaying high tumor burden in mCRPC patient prior to PSMA-RLT. Contrary, PSMA, which has been discussed as a biomarker for PSMA targeted treatment, does not display strong prognostic ability - at least on the mRNA level. Surprisingly, none of these parameters correlates to response to PSMA treatment. In contrast, commom NED markers such as SYP and ENO2 as well as FOXA1 expression level seem to predict OS, but not PFS, more reliably. We admit that a limitation of our study is the focus on mRNA expression of potential biomarkers only. Further investigations analyzing the potential role of protein expression of these markers are therefore warranted.

## Introduction

Prostate cancer (PC) is the most frequent cancer in men worldwide 1. Therapy options for PC have evolved over the last decade since the advent of next generation androgen deprivation therapy (ADT) for metastatic castration resistant PC (mCRPC) by androgen receptor (AR) signaling inhibition (abiraterone and enzalutamide) in addition to taxane based chemotherapy (docetaxel and cabazitaxel), Radium-223 and Sipuleucal-T. Five years ago, ^177^Lu-PSMA-radio ligand therapy (^177^Lu-PSMA-RLT) has been added to the therapy regimen as an alternative treatment option for patients with mCRPC 2. Currently, ^177^Lu-PSMA-RLT is under investigation in several clinical trials 3-8. It has been demonstrated that ^177^Lu-PSMA-RLT is able to prolong life expectation with low incidence of side effects 4, 6, 8, 9. Most studies on ^177^Lu-PSMA-RLT deal with the outcome of this therapy and its combination with other agents such as abiraterone, enzalutamide or chemotherapeutics in retrospective studies. However, in order to understand the efficacy and potential of ^177^Lu-PSMA-RLT, further prospective investigations have to be initiated leading to more insights of PSMA-RLT mechanisms. Additionally, the usage of ^177^Lu-PSMA-RLT is still lacking reliable prognostic biomarkers.

There is evidence that PSMA expression is induced by AR signaling 10-12. Given the strong role of AR signaling in mCRPC, the AR-PSMA axis might be eligible for further mechanistic studies on ^177^Lu-PSMA-RLT. Inhibition of AR by novel hormonal treatment has been described to induce expression of PSMA 13. This effect might become a tool to increase PSMA expression prior to imaging approaches in order to visualize tumor lesions or to sensitize prostate cancer cells to anti-PSMA treatment 14, 15. However, it might also lead to false low or negative results if the AR-PSMA axis has become disfunctional, for example by neuroendocrine differentiation (NED) 16. The mechanisms of AR axis downregulation are incompletely understood and similar to other situations this calls for new biomarkers for better differential diagnosis.

One approach for establishment of novel biomarkers are analyses of circulating tumor cells (CTCs) obtained by liquid biopsy approaches 17. Currently, enumeration of CTCs by the FDA-approved CellSearch^®^ system (Menarini Silicon Biosystems, Bologna, Italy) prior to therapy is recommended by the current Prostate Cancer Working Group (PCWG3) guidelines. 18. These analyses might be used for diagnosis of disease progression by increase of CTC counts or to specifically describe the status of disease prior treatment by determination of favorable or unfavorable CTC counts.

Both AR as well as PSMA are expressed in CTCs of prostate cancer, though expression tends to be heterogeneous between patients and within one single patient 17. The AR splice variant AR-V7 is discussed controversely in predicting worse outcome for AR-V7^pos^ patients 19, 20. What exactly separates AR-V7^pos^ patients from AR-V7^neg^ individuals, however, is poorly reported. An intriguing phenomenon associated with loss of AR signaling is the development of a neuroendocrine signature after tumor progression within the castration-resistant stage. This NED might be connected with loss of AR-V7 expression 16. Once differentiated into a neuroendocrine tumor, common treatment strategies for mCRPC have no more impact and therapy response remain poor if not absent at all and need new therapy options 21. To enable the identification of specific late stage prostate cancer signatures, new diagnostic tracks have therefore to be considered. Using a combinatorial setting of molecular imaging (PSMA PET-CT), clinical parameters as well as novel molecular liquid biopsy parameters (gene expression of AR, AR-V7 or PSMA), CTCs provide a powerful diagnostic tool for patients with advanced mCRPC. The aim of this pilot study was to evaluate the potential value of these parameters in mCRPC patients receiving ^177^Lu-PSMA radioligand therapy.

## Methods

### Patients

Nineteen patients with mCRPC, eligible for ^177^Lu-PSMA-RLT were prospectively enrolled in this study. Inclusion criteria were at least one line of chemotherapy and treatment with either abiraterone or enzalutamide, or both. Blood samples were collected prior to the first cycle of ^177^Lu-PSMA application.

The decision on ^177^Lu-PSMA-617 RLT was individually made in each case by an interdisciplinary tumor board. ^177^Lu-PSMA-617 RLT was offered in case of exhausted recommended therapeutic options and disease progression according to guidelines for mCRPC management 22. All patients received detailed information on the possible adverse events and risks which may be associated with ^177^Lu-PSMA-617 therapy. All patients gave their written informed consent prior to treatment and blood sampling. This study was approved by the local ethics committee (No. 2007-467-f-S and No. 2016-585-f-S, Ethikkommission der Ärztekammer Westfalen-Lippe und der Westfälischen Wilhelms-Universität Münster). Overall survival (OS) was determined as the lifespan in months from the start of PSMA-RLT to death or last contact. PSA decline was calculated as the change of PSA serum levels from baseline compared to the respective time-point.

### CTC enrichment and quantitative real time PCR Analysis

Enrichment of circulating tumor cells as well as analysis of expression of AR, AR-V7 and PSA has been performed as described elsewhere 23. Briefly, peripheral blood collected in EDTA tubes was processed using the Dynabeads™ Epithelial Enrich Kit followed by mRNA isolation using the Dynabeads™ mRNA DIRECT™ Purification Kit (both ThermoFisher Scientific, Waltham, MA, USA). Reverse transcription of mRNA was achieved using the SuperScript™ IV VILO™ Master Mix (ThermoFisher Scientific, Waltham, MA, USA) in a 20µL reaction.

A known disadvantage during the process of CTC enrichment from blood samples is the non-specific capture of leukocytes 24. To specifically determine presence or absence of CTCs, we therefore performed analysis of healthy blood donor samples (n=10) for expression of KLK3-PSA. We did not observe blood cell based expression of KLK3-PSA in any of the healthy blood donor samples. Thus, any KLK3-PSA signal in patient blood samples indicates presence of CTCs. CTCs were detected in 19/19 (100%) of the patient samples. In order to specifiy expression of potential biomarkers to CTCs rather than contaminating leukocytes, healthy blood donor samples (n=10) were processed identical to patient blood samples.

AR-FL, KLK3-PSA, FOXA1, ENO2, SYP and NCAM expression were analyzed using TaqMan assays (AR-full Hs00171172_m1; ENO2 Hs00157360_m1; FOXA1 Hs04187555_m1; KLK3 Hs03063374_m1; NCAM1 Hs00941830; PSMA Hs00379515_m1; SYP Hs00300531) AR-V7 status was analyzed by using a previously described custom-made TaqMan PCR assay (2). All qPCR runs were performed along with TaqMan PCR assays for housekeeping gene RPL37A (Hs01102345_m1) (ThermoFisher Scientific, Waltham, MA, USA).

### CTC specific expression gene expression

Expression levels of biomarker genes for NED (FOXA1, ENO2 and SYP) were analyzed in healthy blood donor samples. In case of expression in healthy individuals, the delta Ct value between marker gene and RPL37A was assesed. The median delta Ct of all healthy controls along with a twofold standard deviation was recalculated to determine a threshold for CTC specific expression levels of NED genes.

Relative copy number levels of AR, AR-V7, PSA, PSMA, FOXA1, ENO2, SYP and NCAM were calculated using the following equation: 2^(42cycles - Ct value). Expression scores were determined using the whole range of relative copy numbers, which were subdivided by logarithmic separation (0-100 copy numbers, score 0 or +; 100-1000 copy numbers, score 1 or ++; etc.). The PSA expression level was used to determine the CTC score, demonstrating a relative level of CTC load. Relative gene expression was determined with PSA gene expression as baseline after normalization of PSA mRNA expression to the housekeeper gene RPL37A.

### Imaging procedures and preparation of ^18^F-PSMA-1007 and image analysis

18F-PSMA-1007 was produced in a GE TracerLab MX synthesizer according to the one-step procedure described by Cardinale et al. and standard operation procedures (SOP), which have been described previously 25-27. Image analysis was performed based on modified PERCIST criteria for 18F-FDG-PET imaging 28. PSMA positive total tumor volume (TTV) was defined analogously to “metabolic tumor volume (MTV)” for FDG-PET imaging according to PERCIST criteria. Treshold-based automatic isoactivity contours were extracted from the complete FoV, using Syngo.via (Siemens Healthcare, Erlangen, Germany). The PERCIST based threshold was defined as 2.0 x SUVmean (mediastinal ROI Aorta) + 2 × SD. Volumes of interest (VOI) contouring noncancer uptake (i.e. kidney, bladder, salivary gland) were deleted manually. Mean SUVmean of the mediastinal blood pool 0.9 ± 0.27. Mean TTV was 744 (range: 20.2 - 3812.7). To investigate the relevance of AR-V7 gene expression for PSMA-PET imaging, the respective images were considered for a visual lesion rating regarding the intensity of PSMA presentation of single lesions. By blinded image rating, PSMA expression was scored into three distinct groups: positive, negative and variable.

### Radionuclide therapy procedures and preparation of ^177^Lu-PSMA-617

The commercially available GMP precursor PSMA-617 (ABX advanced biochemical compounds, Radeberg, Germany) was labelled with ^177^Lutetium (ITG Isotopes Technologies, Garching, Germany) in-house according to radiosynthesis procedures described previously 4, 9. RLT was injected intravenously as a slow bolus in 30 to 60 seconds. Patient had to be hydrated, additional to drinking water, thus receiving at least 1000 mL of Ringer's Solution intravenously following therapy administration to accelerate renal clearance. To reduce uptake in the salivary glands, cooling using standard cooling packs, was started 30 min prior to therapy and maintained for up to 4 h after injection. To confirm ^177^Lu-PSMA-617 uptake and retention in tumor lesions at least one whole-body planar scintigraphy scan and a SPECT/CT scan of the thorax and abdomen was performed 48h after injection. ^177^Lu-PSMA-617 RLT was repeated every 6-8 weeks until progression or death.

### Statistical analysis

Statistical analysis was performed using GraphPad Prism7. The cohort size (n=19) and its subgroups were too small for normality testing. Therefore, all group comparisons were performed by non-parametric t-test (Mann-Whitney). Pearson correlation analysis was performed using the RStudio corrplot package. Kaplan-Meier survival analysis and plotting were performed using SPSS (IBM SPSS Statistics, Version 25.0. Armonk, NY: IBM Corp.). P-values <0.05 were considered statistically noticeable.

## Results

### Patients

A total of 19 patients with a median age of 68.8 years (range 56.9-83.3 years) were selected. All patients had confirmed mCRPC and had underwent previous novel hormonal treatment using abiraterone (94%), enzalutamide (68%) or both (63%). Chemotherapy with docetaxel and/or cabazitaxel was obligatory. Twelve patients (63%) presented with a health status of ECOG 0-1, five patients (26%) with ECOG 2, and one patient (5%) with ECOG 3. The median overall survival of this cohort was 9.9 months (95%CI: 5.4-14.0 months and the median progression-free survival (PFS) was 4.5 months (95% CI: 4.4-4.5) (Figure S1A+B). For more detailed information on patient demographics see Table 1.

All patients displayed the presence of CTCs by KLK3-PSA signals, which demonstrated both a late stage of disease, as well as a high tumor load or metastatic burden.

### Response

**Figure 1:**
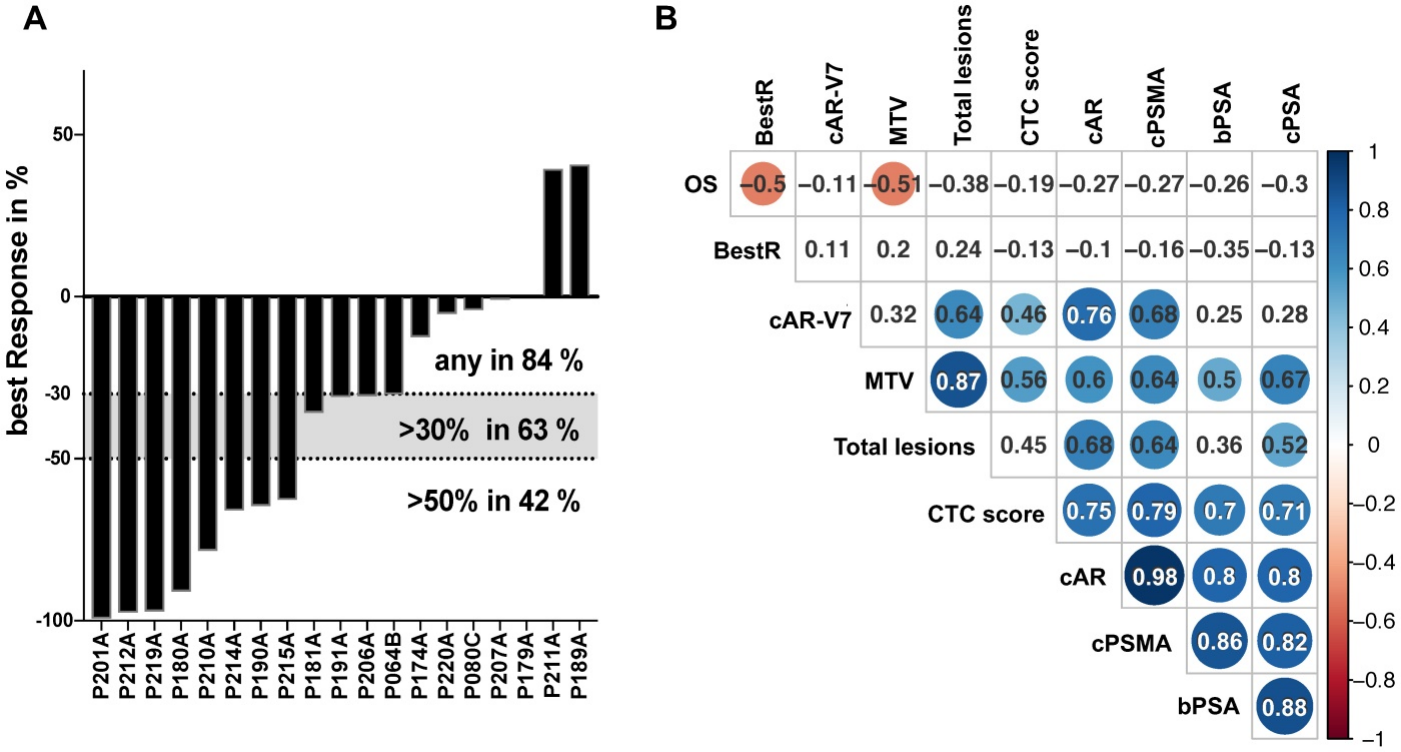
PSA response on ^177^Lu-PSMA therapy and multi-parameter correlation analysis. Best PSA response during therapy with ^177^Lu-PSMA is represented by percentual drop of PSA serum levels and shown in bars for each individual patient in a waterfall plot. Levels >0% represent non-responders, <0% equal any response. Dotted lines indicated established response tresholds of ≥30% and ≥50% (A). Selected parameters have been subjected to a multi-parameter correlation analysis using the RStudio corrplot package. Circle size and colour is linked to amount of correlation (blue for positive, red for negative correlation) and significance (non-significant results with p>0.05 are shown as blank fields). Numbers indicate the precise Pearson value. PSA: prostate-specific antigen; BestR: best response; OS: overall survival; cAR: relative copy numbers of androgen receptor mRNA; cAR-V7: relative copy numbers of androgen receptor splice variant V7 mRNA cPSMA: relative copy numbers of prostate-specific membrane antigen mRNA; bPSA: serum prostate-specific antigen at baseline; CTC: circulating tumor cells; MTV: metabolic tumor volume.

Patients presented with a median PSA serum level of 93.95 ng/mL (range 4.8-4731 ng/mL) at the onset of Lu^177^-PSMA-therapy. Fifteen patients demonstrated a decline of PSA serum levels (84%), 9 patients (63%) presented with a best PSA decline of >30% and 8 patients (42%) with a PSA decline of ≥50% (Figure 1A). The average best response was >30% (-38.2%) and correlated negatively with OS on a low level (pearson coeffeicient 0.5; p<0.05), yet with no other parameters (Figure 1B). Neither a 30% nor a 50% decline correlated with OS (data not shown).

### Multi-parameter correlation analysis

Relative copy number data for CTCs (by PSA score), PSMA, AR and AR-V7 mRNA, baseline serum PSA levels, OS as well as parameters from PSMA PET-CT imaging were subjected to a global correlation analysis (Figure 1B). OS showed correlation to PSA best response (BestR) as well as metabolic tumor volume (MTV) (pearson coeffeicient 0.5 and 0.51, respectively; p<0.05), whereas none of the remaining parameters analyzed in this cohort showed association to OS. The MTV also correlated with total lesions, CTC score, AR, PSMA and PSA relative copy numbers (Pearson coefficient 0.87, 0.56, 0.6, 0.64 and 0.67, respectively; p<0.05), while total lesions correlated with AR and AR-V7 relative copy numbers (0.68 and 0.64, respectively; p<0.05). Both, MTV and total lesions were found to correlate with PSMA relative copy numbers, yet on a medium level (0.64 for both; p<0.05). Baseline serum PSA (bPSA) correlated with MTV, CTC score, AR, PSMA and PSA (0.5, 0.7, 0.8, 0.86 and 0.88, respectively; p<0.05). Progression-free survival (PFS) did not show any correlation with this parameter panel (data not shown).

### AR-V7 subgroup analysis

In a global analysis relative copy numbers of AR and PSMA correlate with tumor load, reflected by MTV and total lesions, which was not detected in case of AR-V7, which correlates to total lesions but not to MTV. To further elucidate the role of AR-V7, the cohort was subdivided into AR-V7^pos^ (n=11) and AR-V7^neg^ (n=8) patients.

**Figure 2:**
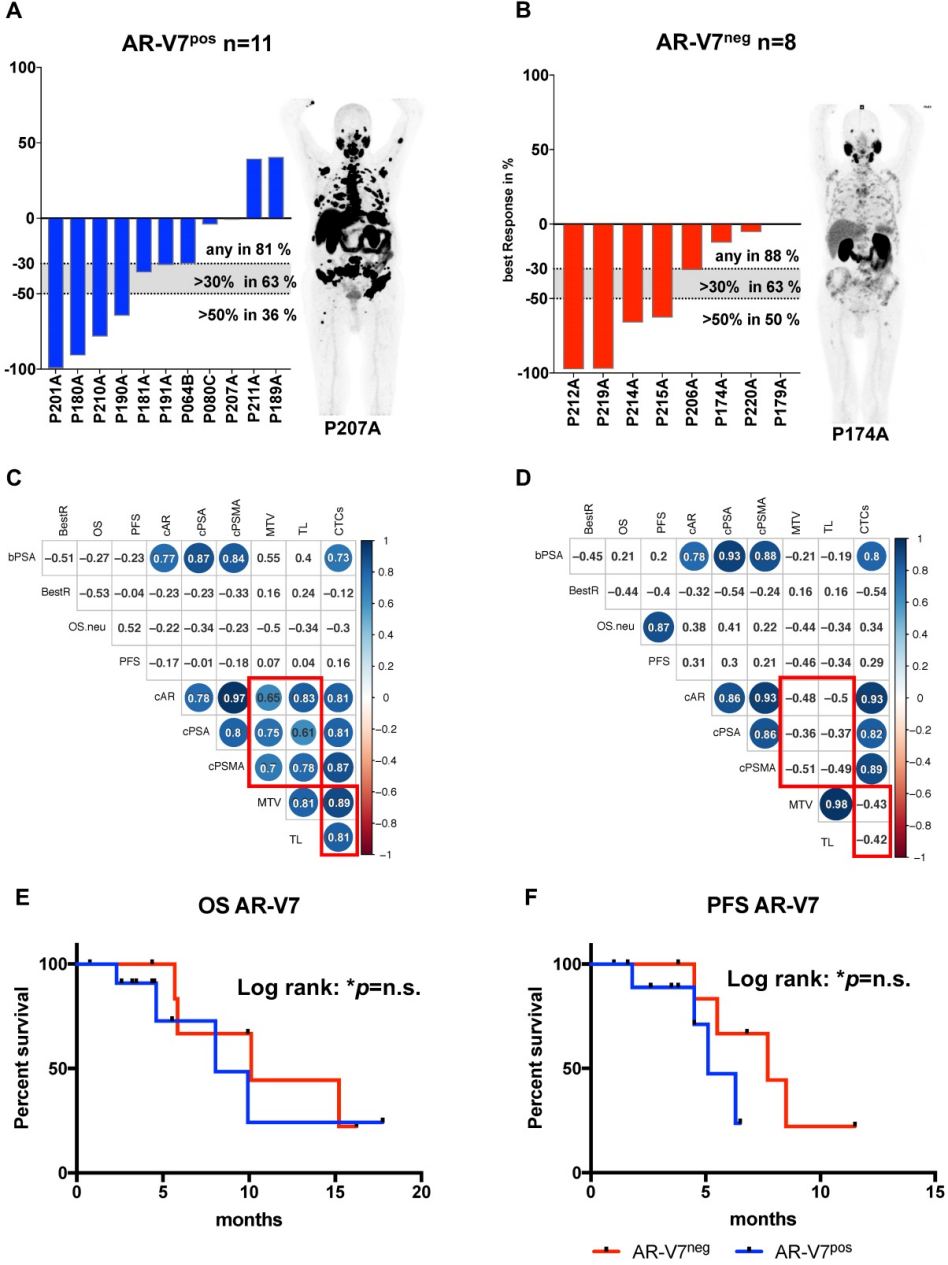
AR-V7 expression subgroup analysis. Best PSA Response is presented for AR-V7^pos^ (A) and AR-V7^neg^ (B) patients. Dotted lines indicated established response tresholds of >30% and >50%. Representative PSMA PET images are shown for each group. Multiparameter correlation analysis is shown in corrplots created using the RStudio corrplot package. Circle size and colour is proportional to the respective Pearson coefficient (blue for positive, red for negative correlation) and significance (non-significant results with p>0.05 are shown as blank fields). Numbers indicate the precise Pearson value. OS (E) and PFS (F) is shown for AR-V7^pos^ (blue) and AR-V7^neg^ (red) patients. PSA: prostate-specific antigen; BestR: best response; OS: overall survival; PFS=progression-free survival; cAR: relative copy numbers of androgen receptor mRNA; cAR-V7: relative copy numbers of androgen receptor splice variant V7 mRNA cPSMA: relative copy numbers of prostate-specific membrane antigen mRNA; bPSA: serum prostate-specific antigen at baseline; CTC: circulating tumor cells; MTV: metabolic tumor volume.

Correlation analysis of these subgroups revealed strong correlation of CTC score, AR and PSMA to MTV and TL (total lesions) for AR-V7 positive patients, while these correlations were absent in AR-V7^neg^ patients (Figure 2C and D). No significant differences were observed in PSA response within these groups. Additionally, within this subgroup, a correlation between PFS and OS was observed, which was not detected within the AR-V7^pos^ subgroup. However, there was no significant difference between AR-V7^pos^ and AR-V7^neg^ regarding OS and PFS (Figure 2 E+F). In a blinded investigation of PSMA-PET images, positive lesions were detected in 72.7% (8 patients) and variable lesions in 27.3% (3 patients) of AR-V7^pos^ patients. AR-V7^neg^ patients displayed variable lesions in 87.5% (7 patients) and negative lesions in 12.5% (1 patient) of this group (Figure S2). AR-V7^neg^ patients presented with a non-significant trend of shorter OS and PFS as well as a missing correlation to PSMA-based imaging results, such as MTV and TL. Given that loss of AR-V7 in late stages of mCRPC might indicate NED, a comprehensive analysis of NED markers was performed. FOXA1 (as a non-NED marker) was detected in patient samples only, whereas SYP and ENO2 expression were also detected in healthy blood donor samples. However, threshold determination revealed a significantly higher expression level in a subgroup of patients (NEDhi) compared to healthy controls (SYP (p<0.0005) and ENO2 (p<0.00005)). Relative copy numbers of FOXA1 were significantly higher within the subgroup of AR-V7 positive patients, whereas no significant differences in relative copy numbers were detected for ENO2 and SYP between AR-V7 subgroups. Nonetheless, patients expressing SYP and ENO2 above threshold had a significantly shorter OS regardless of AR-V7 expression (p<0.05), while this was not observed for PFS. The highest rate of PSMA positive lesions was detected within the AR-V7^pos^ subgroup (8/11 positive, 3/11 variable) by blinded image rating. Contrary, the AR-V7^neg^ subgroup displayed variable or even PSMA negative lesions (7/8 variable, 1/8 negative; Figure S2).

## Discussion

This study, to the best of our knowledge, provides a first clinical and molecular CTC analysis of mCRPC patients prior to Lu^177^-PSMA-therapy.

The membrane spanning PSMA protein has been extensively investigated as a potential biomarker in mCRPC 29-32. However, reports describe a heterogeneous expression pattern in both metastases as well as CTCs 32-34. We detected CTCs in all patients analyzed in this study along with expression of both CTC AR as well as CTC PSMA, although at varying relative expression levels. Thus, expression of PSMA in combination with AR expression in CTCs seems to be a reliable surrogate for the actual tumor load, reflected by MTV and total lesions. These mRNA-based results validate a previous study demonstrating association between Gleason score and PSMA protein expression in grade 1 prostate cancer biopsies 32. Thus, PSMA expression might serve as a clinical biomarker for determination of tumor load.

The AR splice variant AR-V7 is a novel prognostic biomarker for mCRPC. AR-V7 has been reported to predict shorter OS and PFS in patients treated with abiraterone and enzalutamide 20, 35, 36. Contrary however, recent reports have questioned its role as a predictive biomarker given that either AR-V7 positive patients did respond to novel hormonal treatment (NHT) or AR-V7 status was not able to significantly demonstrate shorter OS compared to CTC positive yet AR-V7 negative patients 19, 37-39. AR-V7 expression was detected in 11/19 patients in this study. A correlation between tumor load and higher levels of AR as well as PSMA was found in these patients, whereas this correlation was not detected in AR-V7 negative patients. This observation was further supported by the correlation of CTC score and tumor load. Thus, our results are in line with the recent report by Sharp et al., denominating AR-V7 as a prognostic tool for stratifying patients suffering from a more advanced stage of disease 19.

All studies on AR-V7 display a significant number of patients not responding to antihormonal treatment despite a lack of AR-V7 18, 39, 40. Thus, resistance to these lines of treatment does not necessarily rely on AR-V7 expression. One mechanism, presumably underestimated within mechanistic studies, might be the NED of mCRPC tumors - especially in later stages of disease.

The neuroendocrine state has been characterized by lack of PSMA expression and therefore less effective PSMA-targeted therapies as well as PSMA-supported imaging. However, the onset of NED is difficult to predict and the mechanisms enabling NED are still unclear. NED can be identified by markers, e.g. enolase 2 (ENO2), chromogranin (CgA), synaptophysin (SYP), and CD56 (NCAM) in tissue biopsy staining. In these settings, it is usually accompanied with loss of PSMA and AR expression concomitant with decrease of expression of AR target genes, e.g. FOXA1. 41, 42.

Thus, tumor cells expressing high levels of AR and AR-V7 represent the adenocarcinoma state of prostate cancer, still relying on functional AR axis signaling. In case of mCRPC this might even be accompanied by AR-V7 expression, ultimately regulating AR target gene expression. Yet, absent or low AR levels as well as lack of AR-V7 expression might lead to decrease of FOXA1 expression, which in turn might be indicative for NED 43-45. There is now scientific consensus that NHT might causatively induce NED 46, 47.

In this study, a significant correlation between AR-V7 and FOXA1 expression was detected. Additionally, AR-V7 negative patients displayed significantly increased NED genes, e.g. ENO2, SYP. However, 17/19 (89.5%) and 19/19 (100%) of the patients enrolled in this study displayed AR or PSMA expression, respectively. Given the technical limitations of our approach - not to be able to determine gene expression on a single cell level - some cells might have undergone NED, whereas other cells may still phenotypically reveal an adenocarcinoma stage. Nevertheless, lack of AR-V7 was also accompanied with variable PSMA expression in tumor lesions. Thus, we hypothesize that the loss of AR-V7 might be an indicator for an onset of NED. Expression of SYP and ENO2 in patients as well as in healthy donors does not relate to any the presence nor malignity of the tumor, while FOXA1 seems to be specific for adenocarconima CTCs. We observed a significant drop in FOXA1 levels in AR-V7^neg^ compared to AR-V7^pos^ patients, but no difference regarding ENO2 and SYP. Further, high expression of SYP and ENO2 seems to significantly affect OS, which might be indicative for a more aggressive disease with attributes of NED. To support this hypothesis we included common serum markers such as LDH, ALP and, of course, PSA in our analysis. Significantly lower PSA serum levels in AR-V7^neg^ compared to AR-V7^pos^ patients support the hypothesis of an AR-independent, yet emerging neuroendocrine tumor phenotype 21. While ALP did not show any differences between AR-V7 positive and negative patients, we found higher PSA levels in AR-V7 positive patients and a trend for higher LDH in AR-V7^pos^ patients with low NED gene expression. Higher LDH serum levels have been reported for active tumor metastatic activity and association with shorter OS 48, 49. In our study, elevated levels of the NED genes SYP and ENO2 presented with a significant effect on OS which seemed to function independent from both canonical AR as well as AR-V7 expression. As a result, we conclude that absent AR-V7 expression may be a feature of late stage castration resistance, which contributes to a neuroendocrine stage with elevated levels of SYP and ENO2 as well as a decrease of FOXA1. Additionally, loss of AR-V7 expression might negatively impact PSMA protein expression, given a correlation between absence of AR-V7 and PSMA-based PET imaging. Therefore, PSMA-PET als well as PSMA-RLT have to be critically interrogated in advanced AR-V7^neg^ mCRPC and those patients have to be subjected to a NED-targeted diagnostic panel. Somatostatin-targeting imaging such as 68Ga-DOTATATE-PET and screening for NED biomarkers in CTCs should be considered as clinical options. Using liquid biopsy, changes in gene and protein expression in CTCs can easily be followed. Any changes could subsequently be considered for making reasonable decisions regarding therapy options and changes, thus, rendering PC therapy highly personalized and flexible.

Relative expression levels of cAR, cPSMA and cPSA in CTCs seem to be linked to the actual tumor and metastasis load, represented by their correlation with imaging parameters. In this context, changes of marker gene expression by serial analyses during course of treatment might become a clinical tool of real-time monitoring. Moreover, the effect of ^177^Lu-PSMA-RLT on NED marker expression should be monitored as well. Previous work has identified CTC count during abiraterone treatment as a useful marker for disease progression and survival 50. Further, besides cell count, specific expression level determination of PSMA, AR and AR-V7 is strongly recommended at this point.

On top, analytical and clinical validation has to be performed rigorously before drawing clinical conclusion. We hypothesized PSMA as a potential biomarker for patients which undergo PSMA directed therapy. However, we have to admit, that PSMA expression at baseline does not correlate to response to PSMA targeted therapy. This might rely on detection of PSMA mRNA only without evaluation of functional PSMA protein expression. Thus, future approaches have to focus on detection of PSMA protein rather than mRNA transcripts in CTCs.

Further, according to this study, high transcriptional PSA levels may point at high cAR levels in CTCs. However, serums PSA, as well as its decline, do not display any relation to parameters identified by the molecular analysis of CTCs nor to PSMA-PET-CT imaging data. Therefore, sole PSA response as a predictor might be misleading, as it is not necessarily linked to clinical benefit. Besides, low levels of PSA are also associated with NED 21. A potential synergistic effect of PSA response and decrease of AR as well as AR-V7 expression levels and thus altered AR axis signaling would be an interesting study subject in terms of the induction of NED similar to the effect of a direct androgen deprivation therapy.

Additionally, PSMA- independent imaging methods, such as somatostatin-targeted DOTATATE PET, might give more insights into the phenotypic nature of distinct metastatic lesion and should therefore be included into the diagnostic panel 51.

## Conclusions

Using relative transcriptional levels of novel biomarkers under investigation such as PSMA and AR in liquid biopsies from patients with mCRPC prior to ^177^Lu-PSMA-therapy, this study points at a relation of the molecular diagnostic CTC footprint to tumor load measured by PSMA-PET-CT imaging. However, this approach turned out less useful in terms of prediction of therapy response, survival and time to progression. Therefore, liquid biopsy collection is recommended not only prior to ^177^Lu-PSMA-therapy, but throughout the therapy course in order to detect changes in certain parameter transcription levels. To complete the diagnostic panel CTC proteomics should definitely be included into the analysis. Further, application of PSMA-RLT is questionable in late stage mCRPC, accompanied by loss of AR axis signaling and drop of FOXA1 levels. Also, decreasing serum PSA levels should be considered carefully and viewed in a context with NED markers in order enable distinction between true response and progression to neuroendocrine PC.

## Supplementary Material

Supplementary figures and tables.

## Figures and Tables

**Figure 3:**
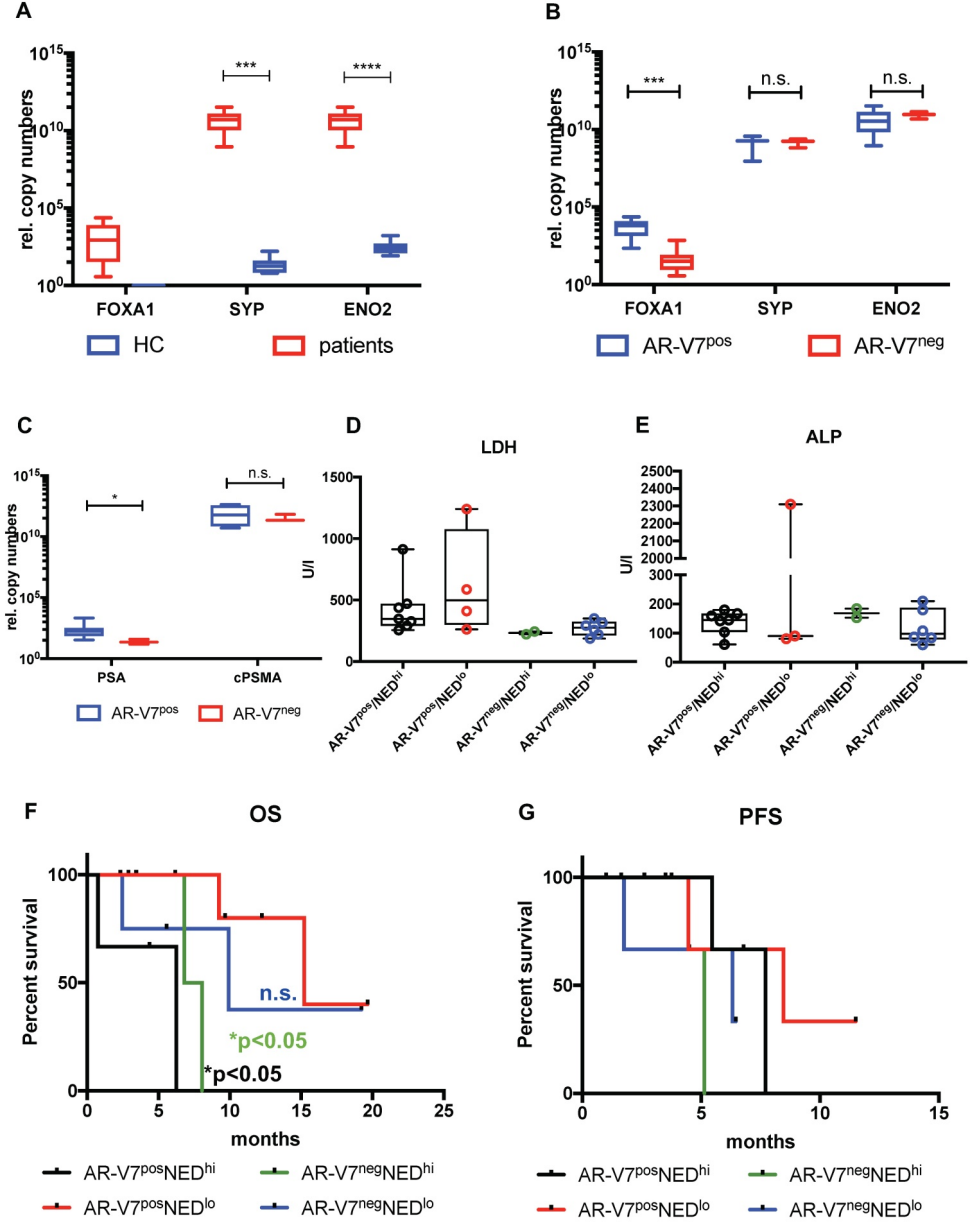
Expression and impact of common NED markers. FOXA1, SYP and ENO2 expression was investigated between healthy controls (HC) and mCRPC patients (A) and between AR-V7^pos^ and AR-V7^neg^ patients (B). FOXA1 expression was normalized to RPL37A, SYP and ENO2 expression was normalized to PSA expression. Normalized expression of FOXA1 was plotted as is, in terms of SYP and ENO2 expression a threshold was determined to separate unspecific expression compared to HCs. AR-V7^pos^ patients were compared to AR-V7^neg^ individuals regarding serum PSA and PSMA mRNA expression (C) ALP and LDH serum values were plotted in a bar plot (D and E). Correspondingly, OS (F) and PFS (G) was determined for four groups: AR-V7^pos^NED^hi^ (black line), AR-V7^pos^NED^lo^ (red line), AR-V7^neg^NED^hi^ (green line), AR-V7^neg^NED^lo^ (blue line). NED: neuroendocrine differentiation; FOXA1: forkhead box A1; ENO2: Enolase 2, SYP: synaptophysin, RPL37A: ribosomal protein 37A; PSA: prostate-specific antigen; OS: overall survival; AR-V7: androgen receptor splice variant V7. P-values (log rank or Mann-Whitney) <0.05 were considered statistically noticeable.

**Table 1:** Patient Characteristics at Baseline (n=19)

Parameters	Median (IQR)
Age (y)	69 (66 -78)
Gleason Score (n=15)	9 (8-9)
PSA (ng/mL)	108 (39-265)
**ECOG**	**N %**
0-1	12 (63)
2	5 (26)
3	1
Alkaline phosphatase (U/L)	142 (81-180)
LDH (U/L)	316 (253-429)
**Site of metastases**	**N %**
Bone	17 (89)
Lymph node	13 (68)
Liver	1 (5)
Lung	1 (5)
Other	4 (21)
**Previous therapy of mCRPC**	**N %**
Docetaxel	15 (79)
Cabazitaxel	5 (26)
Abiraterone or Enzalutamide	19 (100)
Abiraterone	18 (94)
Enzalutamide	13 (68)
Both (Abiraterone & Enzalutamide)	12 (63)
²²³Radium	3 (15)
External-beam radiation therapy to bone	4 (21)
Average number of cycles	2.6 (1-4)
Average dose per cycle	7.64 MBq

PSA: prostate specific antigen; mCRPC: metastatic castration resistant prostate cancer; ECOG PS: Eastern Coloborative Oncology Group Performance Status; LDH: Lactate dehydrogenase.

## Abbreviations

^177^Lu: ^177^Lutetium
PSMA: prostate-specific membrane antigen
PSA: prostate-specific antigen
PET/CT: positrone emission tomography/ computer tomography
AR: androgen receptor
qRT-PCR: quantitative real-time polymerase chain reaction
