# Supplementary material for: Molecular analysis of circulating tumor cells of metastatic castration-resistant Prostate Cancer Patients receiving 177Lu-PSMA-617 Radioligand Therapy
Source: Theranostics. 2020 Jun 18;10(17):7645–55. doi: 10.7150/thno.44556 (PMC7359074; doi:10.7150/thno.44556)
Supplement: Supplementary file 1 — Supplementary figures and tables. [file thnov10p7645s1.pdf]

Supplementary Material

Figure S1

Median survival and progression-free survival. Kaplan-Meier curves are shown for median OS (A) and median PFS (B). OS was determined 9.9 months (95% CI 5.8-14.0 months) and PFS was 4.5 months with 7 death events during the observation period.

Figure S1

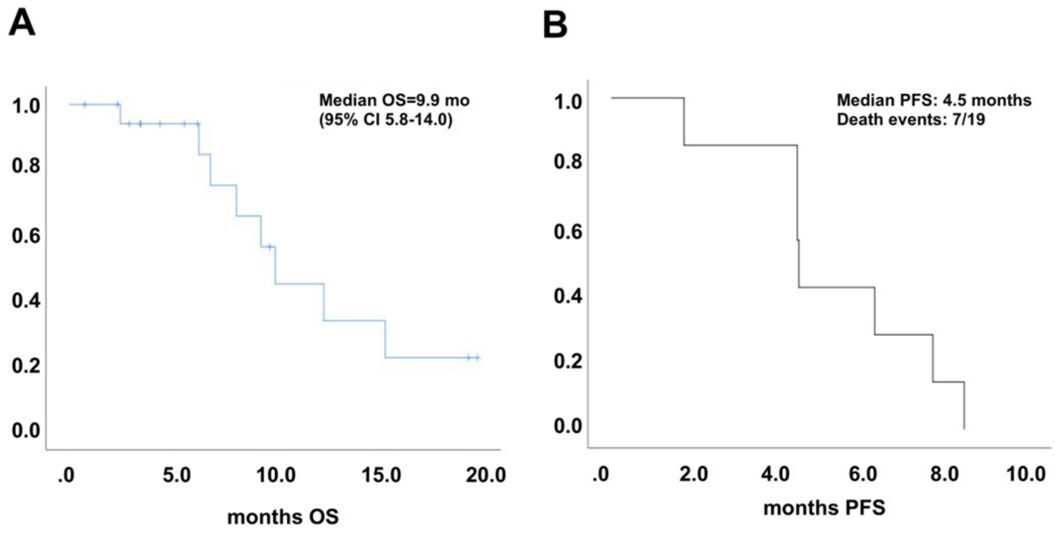

Figure S2:

Blinded evaluation PSMA PET/CT images. Tumor lesions have been classified as PSMA-positive, negative and variable (A). PSMA-PET/CT images have been analyzed and assigned according to this classification for each patient (B). Results were subjected to a grouped analysis for AR-V7^{pos} vs. AR-V7^{neg} patients and plotted in stacked bars per group.

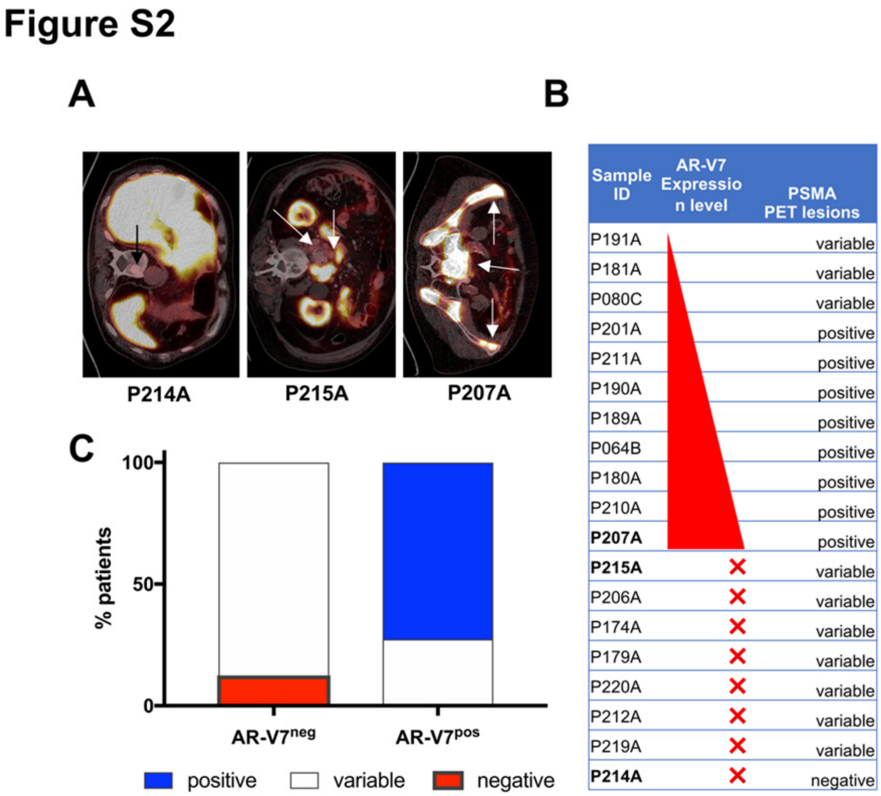

Sample ID	AR-V7 Expression level	PSMA PET lesions
P191A		variable
P181A		variable
P080C		variable
P201A		positive
P211A		positive
P190A		positive
P189A		positive
P064B		positive
P180A		positive
P210A		positive
**P207A**		positive
**P215A**	✕	variable
P206A	✕	variable
P174A	✕	variable
P179A	✕	variable
P220A	✕	variable
P212A	✕	variable
P219A	✕	variable
**P214A**	✕	negative
